# RFX5 promotes the growth, motility, and inhibits apoptosis of gastric adenocarcinoma cells through the SIRT1/AMPK axis

**DOI:** 10.1515/biol-2025-1176

**Published:** 2025-10-08

**Authors:** Lili Zhu, Le Qi

**Affiliations:** Department of Gastroenterology, Anhui Provincial Public Health Clinical Center, No. 100 Huaihai Avenue, Xinzhan District, Hefei, Anhui, 230001, China

**Keywords:** gastric adenocarcinoma, RFX5, SIRT1/AMPK, migration, apoptosis

## Abstract

Gastric cancer is among the most common gastrointestinal malignancies with high morbidity and mortality rates, highlighting the need to further elucidate its pathogenesis and identify effective therapeutic targets. The regulatory factor X (RFX) gene family encodes transcription factors implicated in the development and progression of several cancers. Although RFX5 has been reported to influence tumor progression in various malignancies, its specific role in gastric adenocarcinoma remains unclear. In this study, we investigated the functional effects of RFX5 in gastric adenocarcinoma. Our findings revealed that RFX5 is highly expressed in gastric adenocarcinoma tissues. Silencing of RFX5 significantly inhibited cell proliferation and migration, while promoting apoptosis in gastric adenocarcinoma cells. Mechanistically, RFX5 knockdown activated the silent information regulator transcript 1/adenosine monophosphate-activated protein kinase (SIRT1/AMPK) signaling axis. These results suggest that RFX5 facilitates the growth and motility of gastric adenocarcinoma cells and suppresses apoptosis, at least in part, through modulation of the SIRT1/AMPK pathway.

## 1 Introduction

Gastric cancer remains one of the most prevalent gastrointestinal malignancies in China, with high morbidity and mortality rates [1]. Among its histological subtypes, gastric adenocarcinoma is the most common, accounting for approximately 90% of all gastric malignancies. This subtype originates from malignant epithelial cells exhibiting glandular differentiation of the gastric mucosa, with known risk factors including smoking and heavy alcohol consumption [2]. Although recent decades have seen improvements in early diagnosis and surgical techniques that have reduced overall mortality, the prognosis for many patients remains poor due to advanced-stage diagnosis with metastasis or recurrence at presentation [3]. Accordingly, elucidating the molecular mechanisms underlying disease progression and identifying effective therapeutic targets remain urgent clinical priorities.

The regulatory factor X gene family encodes transcription factors characterized by a highly conserved DNA-binding domain, and previous studies have demonstrated their involvement in cancer development and progression [4]. High expression of RFX4 has been associated with glioblastoma progression [5]. RFX5 has been shown to promote hepatocellular carcinoma (HCC) progression through transcriptional activation of KDM4A [6] and by inhibiting apoptosis [7]. In addition, RFX5 can transcriptionally activate LINC00504 in breast cancer cells, thereby promoting their growth *in vitro* [8]. Furthermore, miR-4319 inhibits lung cancer growth by suppressing YAP expression via regulation of LIN28-mediated stabilization of RFX5 [4]. However, the function of RFX5 in gastric adenocarcinoma remains unclear and requires further clarification. We selected RFX5 as a focus of investigation due to its established oncogenic roles in other malignancies, its overexpression in gastric adenocarcinoma tissues, and the unexplored potential of its mechanistic association with the silent information regulator transcript 1/adenosine monophosphate-activated protein kinase (SIRT1/AMPK) axis in this setting.

The SIRT1/AMPK axis plays a dual role in tumor development and progression. SIRT1 modulates several tumor-related proteins, such as p53 and NF-κB, through deacetylation, thereby influencing cellular apoptosis, inflammation, and metabolism [9,10]. Concurrently, AMPK functions as an energy sensor and can inhibit tumor growth by regulating key pathways such as mechanistic target of rapamycin (mTOR) and fatty acid biosynthesis [11,12].

This study aimed to clarify the role of RFX5 in gastric adenocarcinoma progression and to determine its regulatory interaction with the SIRT1/AMPK signaling pathway, thereby providing insights into its potential as a therapeutic target. We demonstrated that RFX5 promotes the growth and motility of gastric adenocarcinoma cells while suppressing apoptosis, effects that are mediated through the SIRT1/AMPK axis. These findings indicate that RFX5 may represent a novel oncogenic driver in gastric adenocarcinoma and offer a potential molecular target for therapeutic intervention in this disease.

## 2 Materials and methods

### 2.1 Cell Culture

The human gastric epithelial cell line GES-1 and the gastric adenocarcinoma cell lines MKN28, MGC803, and AGS were obtained from the American Type Culture Collection. All cell lines were cultured in Dulbecco’s Modified Eagle Medium supplemented with 10% fetal bovine serum and maintained at 37°C in a humidified incubator containing 5% CO_2_.

### 2.2 Reagents and antibodies

TRIzol reagent was purchased from Invitrogen (15596026). The PrimeScript RT Reagent Kit (RR047A) and SYBR Green PCR Kit (RR820A) were obtained from Takara. RIPA buffer (P0013B), BCA Protein Assay Kit (P0012S), enhanced chemiluminescence (ECL) detection kit (P0018S), CCK-8 kit (C0037), Annexin V-FITC/PI apoptosis detection kit (C1062), crystal violet staining solution (C0121), and Matrigel (BD Biosciences, 356234) for invasion assays were bought from Beyotime.

The following primary antibodies were purchased from Abcam and used at 1:1,000 dilution unless otherwise specified: RFX5 (Ab140621), SIRT1 (Ab110304), phospho-AMPK (Thr172) (Ab133448), total AMPK (Ab32047), BAX (Ab32503), BCL-2 (Ab182858), cleaved caspase-3 (Ab32042), caspase-3 (Ab32351), and GAPDH (Ab8245, 1:5,000).

### 2.3 Quantitative real-time PCR (qRT-PCR)

Total RNA was isolated using TRIzol reagent (Invitrogen, 15596026) following the manufacturer’s instructions. Reverse transcription was performed using the PrimeScript RT Reagent Kit (Takara, RR047A). qRT-PCR was conducted using the SYBR Green PCR Kit (Takara, RR820A). The following primers were used: RFX5: forward 5′-CCGGAAGGAGAGCCTACAGA-3′ and reverse 5′-GGAGTGTCGATGTCGTAGGG-3′; GAPDH: forward 5′-GGAGCGAGATCCCTCCAAAAT-3′ and reverse 5′-GGCTGTTGTCATACTTCTCATGG-3′

### 2.4 Western blot analysis

Protein samples (20–40 µg) were separated by sodium dodecyl sulfate - polyacrylamide gel electrophoresis (10–12%) and transferred onto polyvinylidene difluoride membranes (Millipore, IPVH00010). Membranes were blocked with 5% non-fat dry milk in tris-buffered saline with Tween-20 for 1 h at room temperature and incubated overnight at 4°C with primary antibodies. After washing, the membranes were incubated with appropriate HRP-conjugated secondary antibodies for 1 h at room temperature. Protein bands were visualized using the ECL detection kit (Beyotime, P0018S).

### 2.5 Cell growth assay

Cell viability was assessed using the CCK-8 assay (Beyotime, C0037). Cells were seeded into 96-well plates at a density of 5 × 10^3^ cells per well. After incubation, 10 µL of CCK-8 reagent was added to each well, and absorbance at 450 nm was measured using a microplate reader.

### 2.6 Cell migration and invasion assays

Transwell assays were performed using 24-well Transwell chambers (Corning, 3422) with 8-μm pore size membranes. For migration assays, 5 × 10^4^ cells were seeded into the upper chamber in serum-free medium. For invasion assays, Matrigel-coated Transwell chambers (BD Biosciences, 356234) were used. After 24 h, cells were fixed, stained with crystal violet, and counted under a phase-contrast microscope (Olympus IX73).

### 2.7 Flow cytometry for apoptosis analysis

Apoptosis was assessed using the Annexin V-FITC/PI Apoptosis Detection Kit (Beyotime, C1062) according to the manufacturer’s instructions. Samples were analyzed using a BD flow cytometer (338960).

### 2.8 Statistical analysis

Data are presented as mean ± SD. Statistical analysis was performed using GraphPad Prism 9.0 (GraphPad Software, USA). Student’s *t*-test was used for comparisons between two groups, and one-way ANOVA was applied for comparisons among multiple groups.

## 3 Results

### 3.1 RFX5 is highly expressed in gastric adenocarcinoma

**Figure 1:**
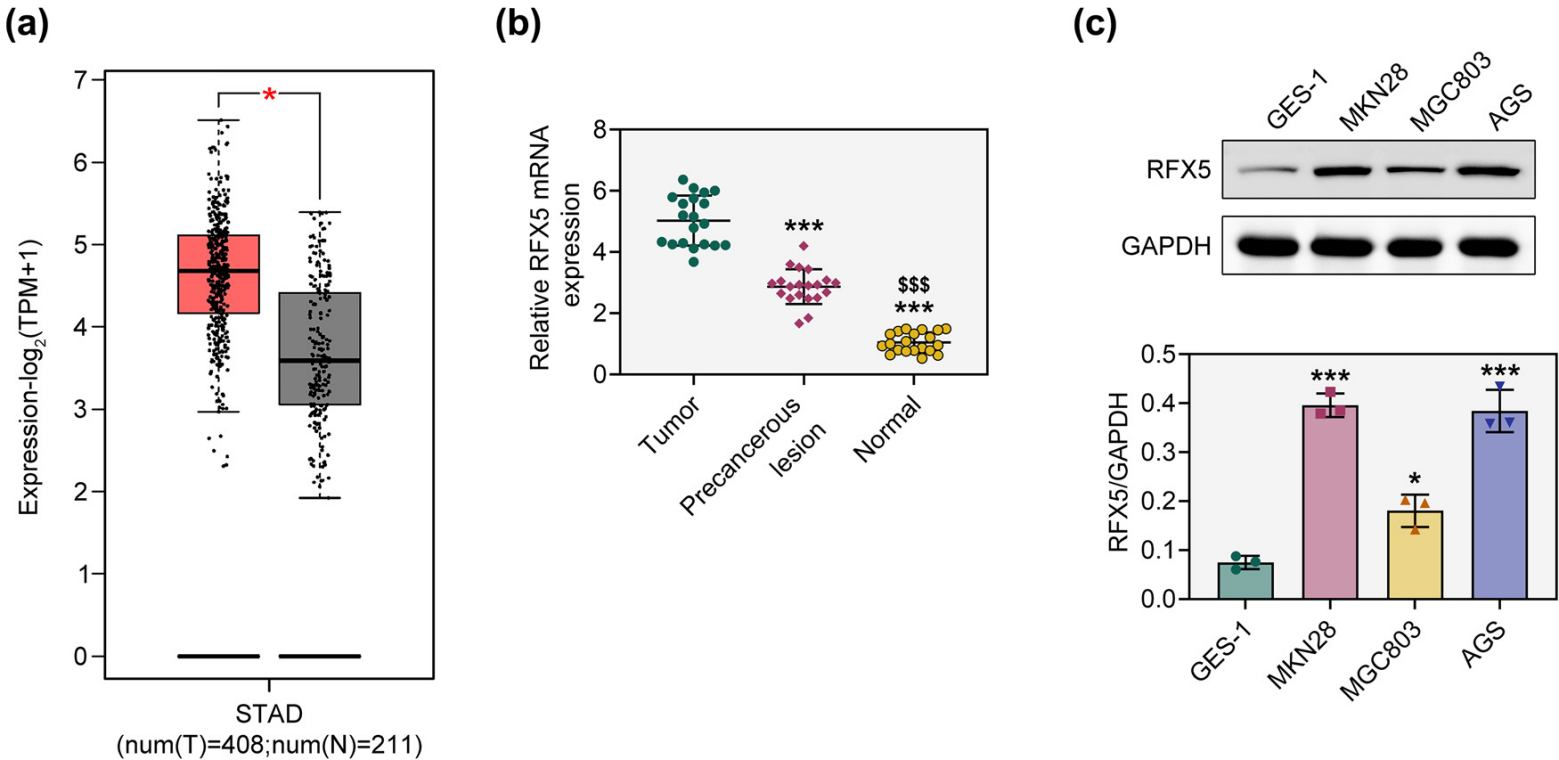
RFX5 is highly expressed in gastric adenocarcinoma. (a) RFX5 expression in stomach adenocarcinoma (STAD) tissues (T, *n* = 408) and normal tissues (N, *n* = 211) was analyzed using the GEPIA database. *P < 0.05. (b) Relative mRNA expression of RFX5 in tumor tissues, precancerous lesions, and adjacent normal tissues was measured by RT-qPCR. ***P < 0.001 vs tumor; ^$$$^ *P* < 0.001 vs precancerous lesion. (c) Western blot analysis of RFX5 protein expression in normal gastric epithelial cells (GES-1) and gastric adenocarcinoma cell lines (MKN28, MGC803, AGS). The lower panel shows densitometric quantification of RFX5 normalized to GAPDH. *P < 0.05, ***P < 0.001 vs GES-1. Data are presented as mean ± SD.

To investigate the expression pattern of RFX5 in gastric adenocarcinoma, we first analyzed its mRNA levels using the GEPIA database. The results showed that RFX5 was significantly upregulated in gastric adenocarcinoma tissues (Figure 1a). To further validate this observation, we performed RT-qPCR analysis on clinical samples, including gastric adenocarcinoma tissues, precancerous lesions, and adjacent normal tissues. Consistent with the database findings, RFX5 mRNA levels were elevated in tumor tissues compared to both precancerous and normal tissues (Figure 1b). In addition, Western blot analysis revealed that RFX5 protein expression was increased in gastric adenocarcinoma cell lines relative to normal gastric epithelial cells (Figure 1c). These results suggest that RFX5 is overexpressed in gastric adenocarcinoma and may be involved in tumor progression.

### 3.2 Knockdown of RFX5 inhibits the growth and migration of gastric adenocarcinoma cells

**Figure 2:**
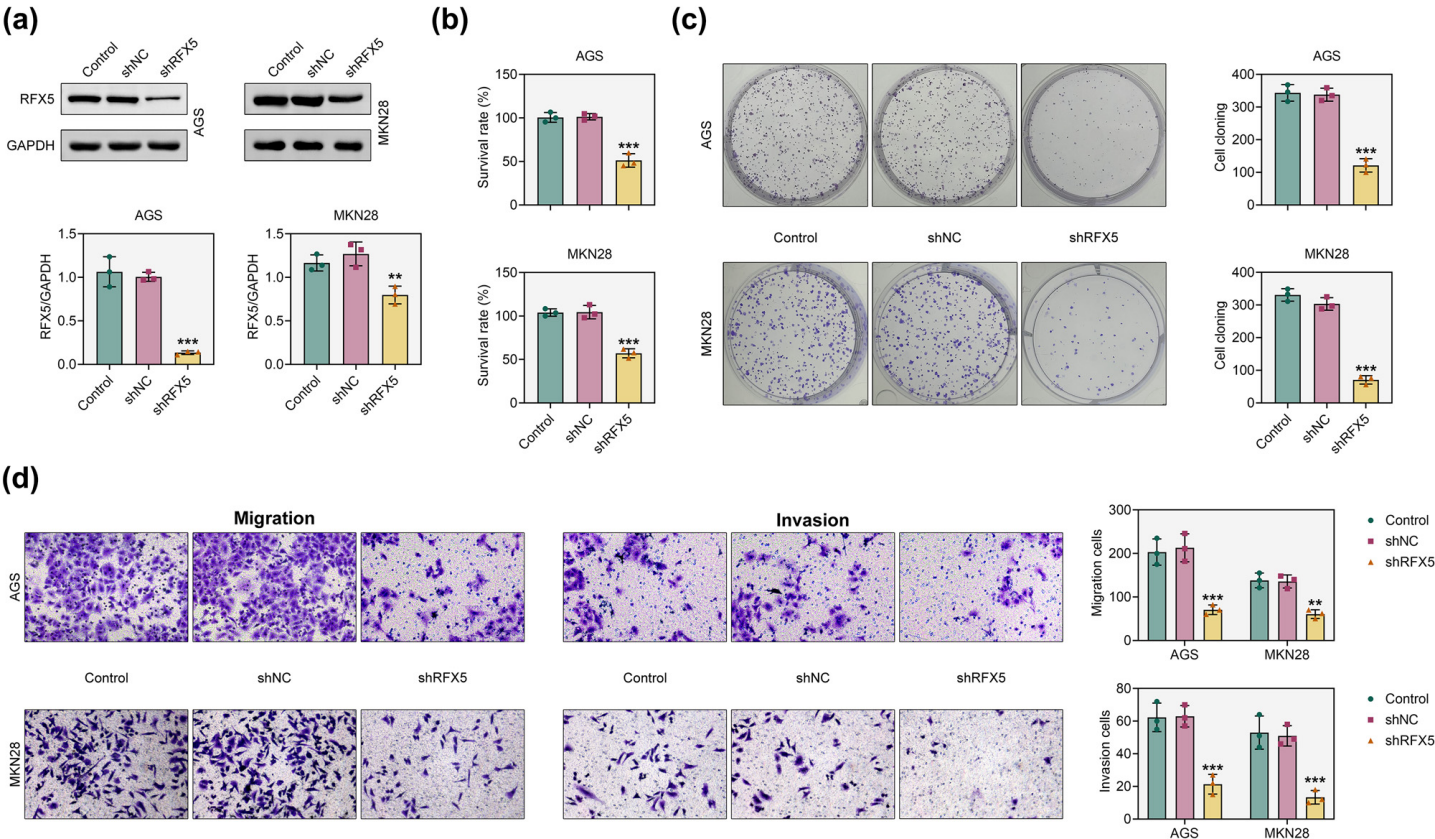
Knockdown of RFX5 inhibits the growth and migration of gastric adenocarcinoma cells. (a) Western blot analysis of RFX5 protein expression in AGS and MKN28 cells following transfection with RFX5-targeting shRNA (shRFX5). GAPDH was used as a loading control. Densitometric quantification of RFX5 expression relative to GAPDH is shown in the lower panel. (b) Cell viability of AGS and MKN28 cells after RFX5 knockdown was assessed using the CCK-8 assay. (c) Colony formation assay evaluating the proliferative capacity of AGS and MKN28 cells following RFX5 knockdown. Representative images are shown on the left; quantification of colony numbers is shown on the right. (d) Transwell migration (left) and invasion (right) assays of AGS and MKN28 cells following RFX5 knockdown. Representative images are shown; quantification of migrated and invaded cells is presented on the right. **P < 0.01, ***P < 0.001 vs shNC.

To explore the functional role of RFX5, we silenced its expression in AGS and MKN28 cells using shRNA (shRFX5). Western blot analysis confirmed effective knockdown of RFX5 protein (Figure 2a). CCK-8 assays demonstrated that RFX5 knockdown significantly reduced cell viability in both AGS and MKN28 cells (Figure 2b). Consistently, colony formation assays showed a marked decrease in the clonogenic capacity of gastric cancer cells following RFX5 knockdown (Figure 2c). To further assess the effect of RFX5 on cell motility, Transwell migration assays were performed. These results indicate that RFX5 silencing significantly reduced the migratory ability of AGS and MKN28 cells (Figure 2d).

### 3.3 Knockdown of RFX5 promotes apoptosis in gastric adenocarcinoma cells

**Figure 3:**
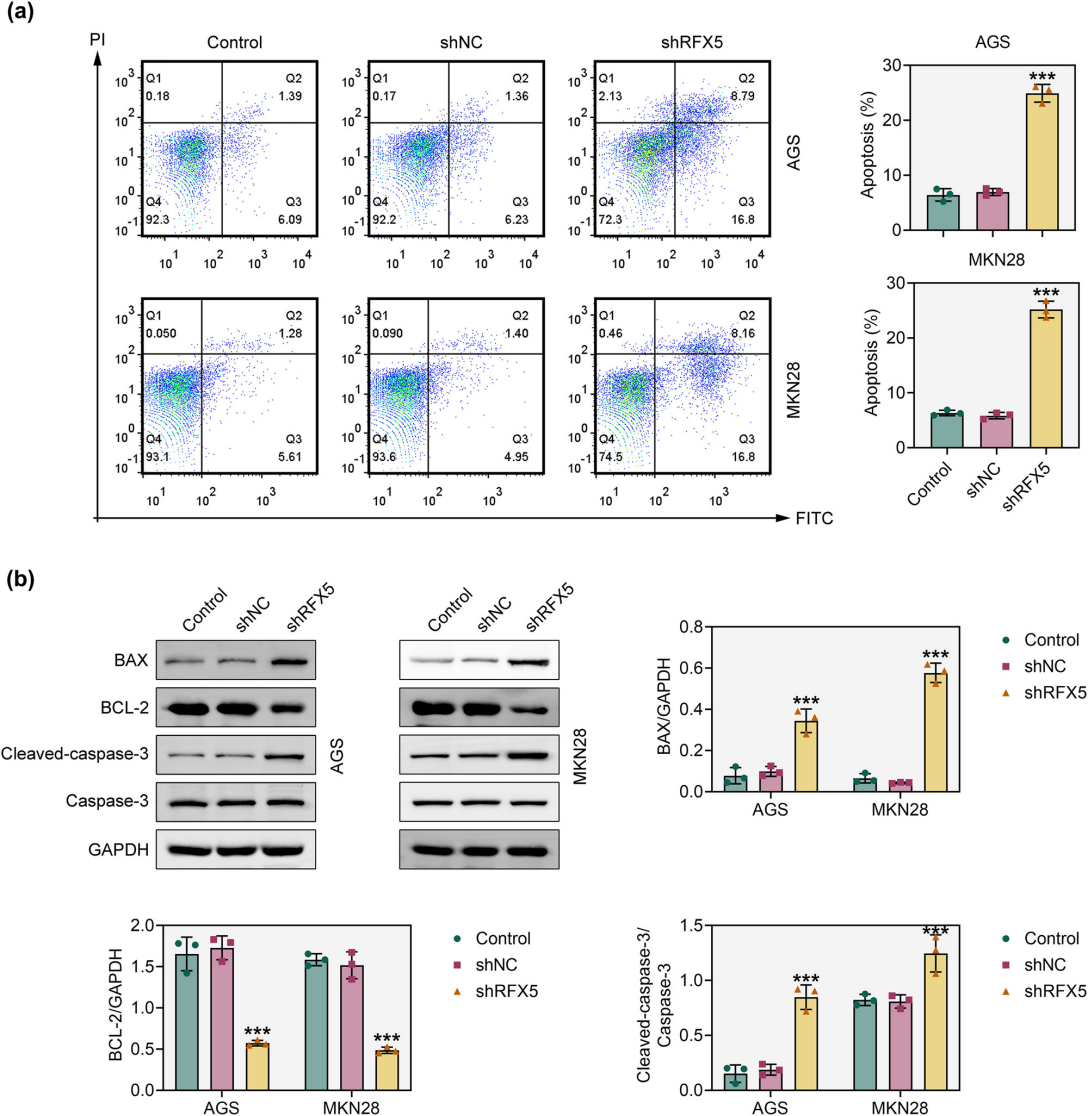
Knockdown of RFX5 promotes apoptosis in gastric adenocarcinoma cells. (a) Flow cytometry analysis of apoptosis in AGS and MKN28 cells following RFX5 knockdown. Representative scatter plots display apoptotic cell distribution in the Control, shNC, and shRFX5 groups. Quantification of apoptotic rates is presented in the bar graphs on the right. (b) Western blot analysis of apoptosis-related proteins, including BAX, BCL-2, cleaved caspase-3, and total caspase-3, in AGS and MKN28 cells after RFX5 knockdown. GAPDH served as a loading control. Quantification of protein expression levels is shown as BAX/GAPDH, BCL-2/GAPDH, and cleaved caspase-3/caspase-3 ratios in the bar graphs. ***P < 0.001 vs shNC.

To determine whether RFX5 influences apoptosis, flow cytometry was performed following RFX5 knockdown. The apoptotic cell population was markedly increased in AGS and MKN28 cells after RFX5 silencing (Figure 3a). Western blot analysis of apoptosis-related proteins further supported this finding. Specifically, RFX5 knockdown led to increased expression of BAX and cleaved caspase-3, accompanied by a decrease in BCL-2 expression (Figure 3b). These results suggest that RFX5 knockdown induces apoptosis in gastric adenocarcinoma cells by modulating key regulators of the apoptotic pathway.

### 3.4 Knockdown of RFX5 activates the SIRT1/AMPK axis

**Figure 4:**
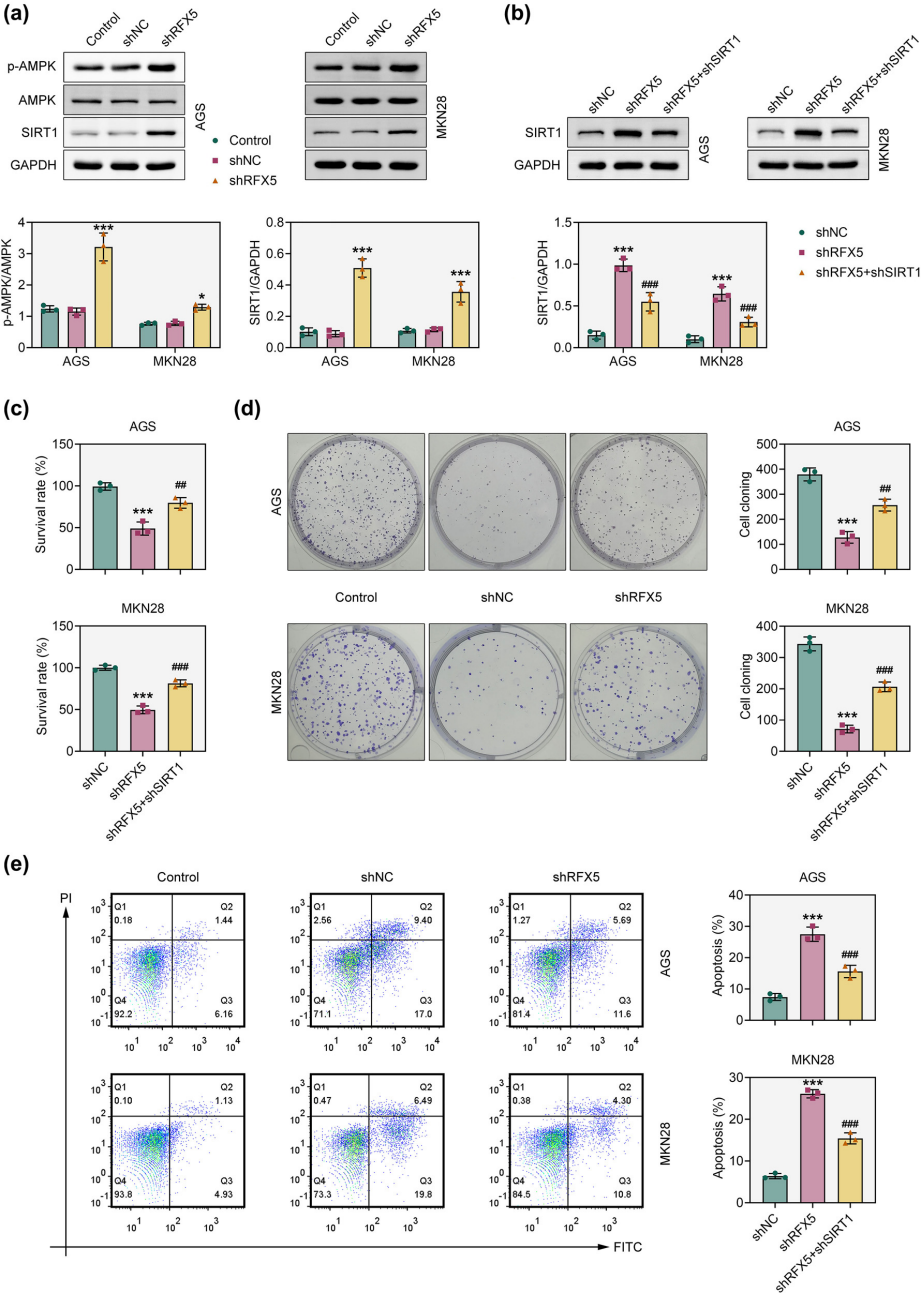
Knockdown of RFX5 activates the SIRT1/AMPK pathway. (a) Western blot analysis of p-AMPK, total AMPK, and SIRT1 protein levels in AGS and MKN28 cells following RFX5 knockdown (shRFX5). GAPDH was used as a loading control. Quantification of p-AMPK/AMPK and SIRT1/GAPDH ratios is shown in the lower panels. (b) Western blot analysis of SIRT1 expression in AGS and MKN28 cells after the indicated transfections. (c) CCK-8 assay assessing cell viability in AGS and MKN28 cells after RFX5 or SIRT1 knockdown. (d) Colony formation assay evaluating the proliferative capacity of AGS and MKN28 cells following knockdown of RFX5 or SIRT1. (e) Flow cytometry analysis of apoptosis in AGS and MKN28 cells following the indicated transfections. ***P < 0.001 vs shNC.

To investigate the molecular mechanism underlying RFX5-mediated tumor progression, we examined its effect on the SIRT1/AMPK signaling axis. Western blot analysis revealed that RFX5 knockdown resulted in increased expression of SIRT1 and elevated levels of phosphorylated AMPK (p-AMPK), while total AMPK levels remained unchanged (Figure 4a). Further analysis showed that the increase in SIRT1 expression following RFX5 depletion could be reversed by co-silencing SIRT1 (Figure 4b). Notably, SIRT1 knockdown also reversed the reduction in cell proliferation and the increase in apoptosis induced by RFX5 knockdown in AGS and MKN28 cells, as demonstrated by CCK-8, colony formation, and flow cytometry assays (Figure 4c–e). These findings suggest that RFX5 negatively regulates the SIRT1/AMPK axis, and its depletion activates this tumor-suppressive pathway in gastric adenocarcinoma cells.

## 4 Discussion

This study demonstrated that RFX5 is highly expressed in gastric adenocarcinoma and that its knockdown significantly inhibits cell proliferation and migration while promoting apoptosis. Moreover, RFX5 depletion led to activation of the SIRT1/AMPK axis, suggesting that RFX5 contributes to tumor progression by negatively regulating this signaling pathway. Collectively, these findings indicate that RFX5 may represent a potential therapeutic target in gastric adenocarcinoma.

The oncogenic role of RFX5 has been reported in several malignancies, including HCC, glioblastoma, and breast cancer [7]. For instance, in HCC, RFX5 has been shown to promote tumor progression through transcriptional activation of KDM4A [13] and to facilitate cell growth in breast cancer cells [14]. However, prior to this study, the role of RFX5 in gastric adenocarcinoma had not been clearly defined. Our results provide new evidence that RFX5 promotes the malignant phenotype of gastric cancer cells and exerts these effects via modulation of the SIRT1/AMPK axis. This finding expands the known oncogenic functions of RFX5 and highlights its potential tissue-specific regulatory mechanisms in cancer.

The SIRT1/AMPK axis is recognized for its context-dependent role in tumor biology, functioning either as a tumor suppressor or promoter depending on the cellular environment [15,16]. SIRT1 regulates several tumor-associated proteins, including p53 and NF-κB, thereby modulating apoptosis, inflammation, and metabolic processes [17]. AMPK, as a key energy sensor, governs cell proliferation, autophagy, and metabolic stability by modulating downstream pathways such as mTOR and lipid biosynthesis [18,19]. Previous studies have indicated that activation of the SIRT1/AMPK axis can suppress tumor growth through induction of apoptosis and cell cycle arrest [20,21]. Our findings support this interpretation, as RFX5 knockdown resulted in increased SIRT1 expression and AMPK phosphorylation, indicating that RFX5 suppresses this tumor-inhibitory pathway to facilitate gastric cancer progression. These results suggest that targeting RFX5 may represent a promising therapeutic approach to reactivate SIRT1/AMPK signaling and suppress tumor growth in gastric adenocarcinoma.

Given the high invasiveness and recurrence rates associated with gastric adenocarcinoma, the identification of novel therapeutic targets remains critically important. The observed upregulation of RFX5 in gastric cancer tissues, along with its regulatory influence on the SIRT1/AMPK signaling axis, suggests that RFX5 may function not only as a diagnostic biomarker but also as a potential therapeutic target. Pharmacological inhibition of RFX5 could offer a promising treatment strategy, particularly for patients exhibiting elevated RFX5 expression. Furthermore, therapeutic approaches aimed at activating the SIRT1/AMPK pathway may be employed in combination with standard therapies to improve clinical outcomes. As RFX5 knockdown leads to SIRT1/AMPK pathway activation, which is associated with suppressed cell proliferation and enhanced apoptosis, the development of RFX5-targeted agents, including small-molecule inhibitors or RNA-based therapeutics, warrants further investigation for potential clinical application.

There are several limitations that should be acknowledged. First, most of our experiments were conducted *in vitro*, and further *in vivo* validation using appropriate animal models is necessary to confirm our findings. Second, although we demonstrated that RFX5 modulates the SIRT1/AMPK signaling axis, it remains unclear whether additional pathways contribute to its tumor-promoting effects. Further investigations are warranted to elucidate potential downstream effectors or cooperative signaling mechanisms involved in gastric cancer progression. Third, our analysis was based on a relatively small cohort of clinical samples, which may limit the generalizability of the results. Therefore, larger-scale clinical studies are needed to determine whether RFX5 expression is significantly associated with patient prognosis and disease severity.

Future research should aim to further elucidate the mechanisms through which RFX5 contributes to tumor progression in gastric adenocarcinoma. Specifically, it will be important to investigate whether RFX5 regulates additional cancer hallmarks, such as angiogenesis, metabolic reprogramming, and immune evasion [22,23]. Additionally, studies assessing the correlation between RFX5 expression and patient survival outcomes may offer valuable prognostic insights. The development of preclinical models to evaluate the therapeutic efficacy of RFX5-targeted interventions will be essential for advancing translational applications. Moreover, considering that RFX5 interacts with multiple signaling pathways, combination strategies targeting both RFX5 and the SIRT1/AMPK axis should be explored to assess potential synergistic effects in tumor suppression. Finally, *in vivo* validation using animal models will be necessary to confirm these findings and investigate the therapeutic potential of targeting RFX5 with SIRT1/AMPK modulators for gastric adenocarcinoma treatment.

In conclusion, this study provides the first evidence that RFX5 promotes the proliferation and migration of gastric adenocarcinoma cells while suppressing apoptosis, primarily through inhibition of the SIRT1/AMPK signaling axis. These findings offer new insights into the molecular mechanisms underlying gastric adenocarcinoma progression and highlight RFX5 as a potential therapeutic target for future clinical intervention.
